# Second-Generation Wide-Field Visualization Devices for 5-ALA–Induced Fluorescence and Concepts for Validation in Neurosurgery—A Systematic Review

**DOI:** 10.1227/neuprac.0000000000000059

**Published:** 2023-10-06

**Authors:** Zeynep Özdemir, Eric Suero Molina, Sönke J. Hellwig, Herbert Stepp, Walter Stummer

**Affiliations:** ‡ Department of Neurosurgery, University Hospital of Münster, Münster, Germany;; § Laser-Forschungslabor, LIFE Center, University Hospital, LMU Munich, Munich, Germany

**Keywords:** 5-ALA, Fluorescence-guided resection, Fluorescence-guidance, Device validation, Filter system

## Abstract

**BACKGROUND AND OBJECTIVES:**

Fluorescence-guided resection (FGR) of malignant gliomas with five-aminolevulinic acid (5-ALA) is an established method using surgical microscopes equipped with filter systems for observing fluorescence. Over the past decade, new technologies have been introduced for the same purpose, with available publications evaluating their clinical efficacy based on varying criteria. This study aims to review technologies and concepts of validation in the context of 5-ALA–mediated FGR.

**METHODS:**

A systematic review following the Preferred Reporting Items for Systematic Reviews and Meta-Analyses statement was performed to identify devices capable of detecting 5-ALA–induced fluorescence. Articles found eligible for this review were analyzed, focusing on the methods of validation used for novel devices. A qualitative analysis is presented.

**RESULTS:**

Using predefined eligibility criteria, 22 studies were analyzed. Publications on the following visualization devices were reviewed: FL400 (Leica Microsystems), Aeos (Aesculap), BLUE400 and BLUE400 AR Filter System (Carl Zeiss Meditec AG), Endoscope with D-Light C (Karl Storz), Fiberscope N-4L (Machida), ORBEYE 4K 3D Digital Video Microscope (Olympus), and several customized surgical loupe systems. In many cases, validation seemed unstandardized, with inherent biases and limited reproducibility.

**CONCLUSION:**

This review illustrates the significance of device validation within the framework of FGR. It emphasizes the criticality of validating devices in accordance with established standard, i.e. the BLUE400 filter system, which was employed in the approval studies of 5-ALA. Furthermore, standardized concepts of validation are required to assess whether new devices are, in fact, a reliable or superior alternative in the field of FGR. Published guidelines should be considered when performing future studies.

**ABBREVIATIONS:**
5-ALA: five-aminolevulinic acid
FGR: fluorescence-guided resection
NPV: negative predictive value
PpIX: protoporphyrin IX.

Five-aminolevulinic acid (5-ALA) is approved for fluorescence-guided resection (FGR) of high-grade glioma.^1,2^ It is based on selective accumulation of protoporphyrin IX (PpIX), a natural porphyrin in the heme biosynthesis, in glioma tissue after oral 5-ALA administration. Fluorescence is directly made visible to surgeons with the help of optical filters.^2^ Using blue/violet light in the wavelength of 375 to 440 nm and specific emission filters, which are incorporated in the operative microscope, PpIX fluorescence is made visible. Thus, FGR allows a greater extent of resection, which is associated with longer overall survival rates and progression-free survival.

The pivotal phase III approval study was performed exclusively using modified surgical microscopes with filtered xenon excitation light and specific detection filters,^3-5^ a technology allowing direct perception of fluorescence without intervening video cameras or image processing. A defined degree of excitation light provides background illumination and, importantly, cloak weak but omnipresent red autofluorescence from intrinsic fluorochromes, such as NADH, lipofuscin, and flavins, but possibly also weak PpIX fluorescence from infiltrating tumor cells.^4,6-8^ The filter combination, therefore, defines a threshold of sensitivity for detecting tumor cells with a high degree of specificity,^3,4^ exceeding the volume of gadolinium enhancement on MRI.^9-11^ Modified cameras allow video imaging. However, whether resulting images authentically reproduce fluorescence directly observed by surgeons is unclear.

## The Necessity for Testing Novel Devices

Because approval studies for FGR relied on specific excitation light/filter combinations, we assume that new technologies require testing to ensure specificities and sensitivities comparable with the established standard. Less sensitive systems would underestimate tumor while greater sensitivity may cost specificity, possibly resulting in over-resection.^12^ Newer technologies might help overcome limitations of standard fluorescence microscopes, such as long working distances and weak illumination brightness.^13,14^ Novel technologies such as multispectral or hyperspectral imaging^15^ might also aim at overcoming the interference by red light components of autofluorescence and ambient light, which contaminates PpIX fluorescence. Under certain conditions, tissue will backscatter the red fraction predominantly from white ambient light, producing a false-positive fluorescence impression.

Available new technologies include loupe/diode devices, endoscopy-guided surgery, and exoscopes (ie, extracorporeal telescopes).^16-18^ The latter systems rely only on cameras and video chains with a degree of image processing,^16^ and it is unknown whether these technologies show fluorescence comparable with the established microscopes. Our preliminary experience (Figure) suggests differences between devices. It is also unknown how different light sources might affect phenomena such as photobleaching.^2,4^

**FIGURE.**
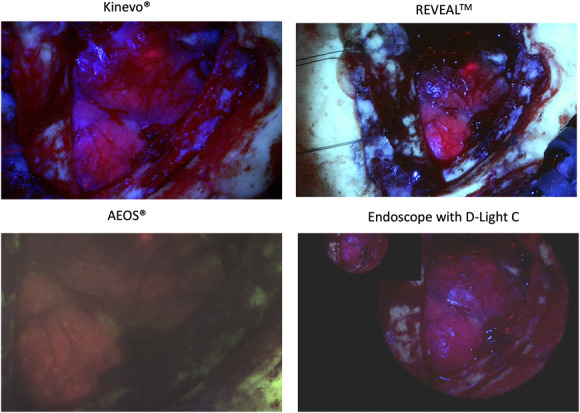
Images from the cortical surface of a patient with glioblastoma. Note differences in fluorescence imaging, background depiction and brightness, and fluorescence borders. Top left: Kinevo 900, Carl Zeiss Meditec AG; top right: REVEAL FGS, Designs for Visions; bottom left: AEOS, Aesculap; bottom right: Photodynamic Diagnostics, Karl Storz—Endoscope.

Searching the literature, we only find rare investigations comparing novel technologies with the established standard or using neuropathological validation of visible fluorescence. Uniform assessment criteria need to be used.

However, several criteria can be envisioned, which serve to characterize an imaging device (Table 1), eg, the technical basis of imaging (direct fluorescence observation vs detection by cameras only); choice of excitation/emission filters; background illumination modes; whether devices are validated based on reproducible criteria; bleaching; and ease of use, including assistant/observer integration.

**TABLE 1.** Assessment Criteria for Intraoperative Fluorescence Detection

Criterion	Variants	Explanation
Technological platform and light translation	• Filtered red fluorescence directly perceived by the surgeon using a lens magnification system (eg, Blue400, Reveal loupe system) • Filtered red fluorescence captured by a video camera and projected onto a screen aiming at life-like reproduction of fluorescence comparable with Blue400 (Exoscopes, Endoscopes) • Filtered red fluorescence captured by a video camera and enhanced (exoscopes) • Imaging based on spectrographic unraveling of the PpIX tissue spectrum (under development)	• This distinction is pertinent because video chains with or without image processing are susceptible to red and blue light sensitivity of cameras and any image processing for fluorescence enhancement.
Choice of excitation wavelength and emission filter characteristics	• Devices with background illumination (microscopes, some exoscopes, and loupes) • Devices without background illumination (some exoscopes, depending on settings); imaging depending on spectrographic unraveling	• Owing to the characteristics of the PpIX and autofluorescence spectra, the choice of excitation light wavelengths and detection filters will directly influence the sensitivity and specificity of detection as well as the intensity of PpIX fluorescence.^6^
Fluorescence excitation and background illumination intensity	• Various devices are characterized by differences in illumination intensity, derived from xenon or diode illumination	• The background illumination intensity will directly affect background brightness and the relative perceived brightness of fluorescence. Precise adjustment of background illumination intensity plays an important role for methods with pure visual detection. Image adjustment or manipulation using video chains offer freely adjustable background brightness and thus PpIX fluorescence recognition thresholds. • Background illumination and fluorescence excitation might interact; more background illumination could cloak weak fluorescence. • Excitation light intensity correlates with photobleaching.
Validation	• Validation based on histology • Validation based on visibility of fluorescence	• Histological diagnostic accuracy based on reproducible, unbiased sampling algorithms (specificity, sensitivity, NPV, and PPV) is crucial. • Assessing sensitivity of filter systems in comparison with the established standard (BLUE400 or analogous devices).
Surgical ergonomics	• Microscope: Most of the light is directed to the surgeon as opposed to the assistant • Exoscope: Fluorescence is visualized on a video screen and is observed by all participants equally (surgeon, assistant, and nursing staff) • Loupe systems: Fluorescence is only observed by the surgeon	• Ease of use • Observer/Assistant integration • Setup and execution of fluorescence

NPV, negative predictive value; PpIX, protoporphyrin IX; PPV, positive predictive value.

This study aims to define factors characterizing the usefulness of devices for FGR and systematically reviews available publications regarding devices and their performance.

## METHODS

### Assessment Criteria

To analyze articles concerning their evaluation of devices for intraoperative fluorescence detection, we defined assessment criteria based on the previously published Standards for reporting of Diagnostic Accuracy - Central Nervous System (STARD-CNS) criteria (Table 1).^19^ We focused on reported validation criteria, such as histology and device comparison. We hereafter analyzed the validation criteria of the published work regarding possible bias. We used these as a basis for our assessment, including illumination and detection types, validation methods, photobleaching measurements, or observer/assistant integration.

### Literature Search

A MEDLINE search of titles and abstracts was performed using the following search operators on June 2nd, 2022: 5-ALA/5-aminolevulinic acid/ALA/PPIX/Protoporphyrin IX, each in combination with Device/Instrument(s)/novel/exoscope/endoscope/endoscopic/filter/microscope/camera/intraoperative visualization/fluorescence visualization and Endnote X9 (Clarivate Analytics) as the search engine. A second Scopus search was performed on May 1, 2023. Non-English studies, duplicates, and studies on experimental devices were excluded. Two reviewers independently performed the search (ZÖ and ESM) according to the Preferred Reporting Items for Systematic Reviews and Meta-Analyses statement.^20^ The review was not registered in the PROSPERO database because it represents a literature review with a systematic search, which cannot be accepted as to their current guidelines.

## RESULTS

We identified 7270 studies published from 1950 to 2022. After removing duplicates, non-English articles, and studies without abstracts, the titles and abstracts of the remaining 5351 publications were screened (see **Supplemental Figure 1**, http://links.lww.com/NEUOPEN/A70). Video reviews were not included. Articles were excluded if the following criteria were unmet: visualization device, intraoperative fluorescence, 5-ALA, and neuro-oncology. Full-text articles from 43 studies were further assessed. Finally, 22 studies were included for qualitative analysis, as summarized in Table 2 (see **Supplemental Figure 1**, http://links.lww.com/NEUOPEN/A70). This study focuses on 5-ALA–induced fluorescence visualized by novel devices. We did not distinguish between tumor entities because the focus was on the fluorescence signal and its comparability to the established gold standard.

**TABLE 2.** Devices' Excitation Light and Built-in Filters

Device	Company	Excitation light	Emission filters
ORBEYE^31,33^	Olympus, Tokyo, Japan	LED 405 nm	n.a.
Aeos^21,40^	Aesculap, Tuttlingen, Germany	390-420 nm	510 nm LP
Blue400 AR^12^ and pers. Comm.	Carl Zeiss Meditec, Oberkochen, Germany	380-430 nm and orange (wavelength n.a.)	440 nm LP

Endoscope^23^	Karl Storz, Tuttlingen, Germany	380-430 nm	n.a.
HD-Xoscope^28,29^	Karl Storz, Tuttlingen, Germany	380-450 nm	n.a.
Kinevo 900^18^	Carl Zeiss Meditec, Oberkochen, Germany	380-430 nm and blue 450 nm	440 nm LP
REVEAL^18^	Designs for Vision Inc, New York, USA	409 ± 7 nm and blue 450 nm	435 nm LP

Devices' excitation light and built-in filters as presented in the literature. The terms “and blue” or “and orange” imply that a well-adjusted low intensity of such light is additionally illuminating tissue in the transmission wavelength range of the emission filter to serve as a background “ambient light” and to establish a color contrast of red PpIX fluorescence vs the surrounding tissue.

In most reviewed articles included in this study, the time of 5-ALA application was disclosed, which was, as per indication, 2 to 4 hours before surgery.

### BLUE400 (Carl Zeiss Meditec AG)

The oldest filter system has filtered xenon light (375-410 nm for excitation, >425 nm for emission). In an early study using spectrometry and a defined biopsy algorithm, the positive predictive value (PPV) was reported as 98%, specificity 75%, sensitivity 70%, and negative predictive value (NPV) 28%.^7^ Tumor cell densities were correlated with spectrographic fluorescence. BLUE400 is 510(k)-cleared, referring to medical devices approved by the Food and Drug Administration through the 510(k) premarket notification process.

### Leica FL400 (Leica Microsystems)

This device is equivalent to BLUE400 regarding excitation light and emission filters and was validated in a prospective study using biopsies (3-5 per patient) with a PPV of 95% and sensitivity of 97%.^5^ This device is also 510(k)-cleared.

### Aeos (Aesculap)

Aeos is a robotic-assisted exoscope with a 3D 4K monitor, equipped with a DUV400 filter for FGR (excitation wavelength from 390 to 420 nm, visualization longpass filter >510 nm). White light can be mixed into the fluorescence image to enhance background illumination. The device features an augmented reality option.^21^ According to technical details (Table 2), it seems that the contrast to normal tissue is not achieved with blue emission (which corresponds to the 510-nm longpass filter), but rather with green autofluorescence. Thus, this device seems significantly more sensitive (potentially less specific) than other available technologies.

Apart from providing information on the device's usefulness during predefined surgical tasks,^22^ one study compared intraoperative fluorescence (3 patients with glioma) with an OPMI Pentero (CZM) system, claiming a lower fluorescence intensity.^22^ This assessment was based on “relative fluorescent units,” which remain unclearly defined.^22^

### BLUE400 AR Filter System (CZM)

One study compared BLUE400 AR, a technology to improve background discrimination, with BLUE400.^12^ BLUE400 AR had a similar excitation range (400-410 nm), which “also included other discrete spectral components to allow better background illumination,” as stated by the authors. Thus, non-fluorescing tissue appeared yellow-green instead of blue, and fluorescing tumor had an orange shade.^12^ Neuropathological assessment^12^ demonstrated visible fluorescing tumor margins to be comparable using a reproducible biopsy algorithm similar to an older publication.^12^ Fluorescence visualization was superior to that of the BLUE400 filter system, with hemostasis achievable using the fluorescence mode.^12^

### Endoscope D-Light C (Karl Storz)

Four studies focus on an endoscope/D-Light C combination (Karl Storz, excitation light 380-430 nm), including 1 case report.^23^ One small study (n = 9 cases) reported additional fluorescence to be observable, with histological confirmation in 8 of 9 cases.^24^ In 2 other studies on glioblastomas and metastases,^25,26^ additional fluorescence was detected in all cases and was verified as tumor histopathologically.^26^ In brain metastases, the authors reported detecting additional fluorescence tissue using the endoscope in 84.6% of cases, with a sensitivity of 95.5% and specificity of 75%.^25^ Strickland et al^27^ reported endoscopic FGR to allow more significant resection volumes in 83.3% of cases.

### Fiberscope N-4L (Machida)

Tamura et al^17^ reported using a customized fiberscope with ultraviolet laser light (emission wavelengths 405 nm) and a cutoff filter^14,17^ in a patient with an intraventricular lesion^14^ stating the device to show more red fluorescence and allowing a more controlled biopsy.

### HD-Xoscope (Karl Storz)

The HD-Xoscope is a rigid lens telescope first described in 2008 with a blue light filter (excitation wavelength 400-410 nm).^28^ In 15 of 21 malignant glioma cases, total resection (≥95% of the initial volume) was achieved.^28^ Fluorescence was correlated with histopathology.^28^ In a subsequent report (30 craniotomies, 8 biopsy cases), complete resection was achieved in 23 of 30 cases,^29^ with the authors reporting a sensitivity of 73%, specificity of 100%, NPV of 63%, and PPV of 100%.^29^

### Kinevo 900 (Carl Zeiss Meditec AG)

In a laboratory investigation, the Kinevo 900 (CZM) was compared with the CZM Pentero 900,^30^ using tris-mono-europium phantom and in mouse tumors.^30^ The Kinevo 900 showed no differences in the generated fluorescence. However, the authors stated that surrounding, non-fluorescing tissue could be better discriminated.^30^

### ORBEYE 4K 3D Digital Video Microscope (Olympus)

The ORBEYE is a 4K 3D semirobotic exoscope with 3 imaging modes (infrared, blue, and narrow-band) with blue LEDs for porphyrin excitation.^31^ Because emitted light is imaged onto a video chip, lower light conditions seem sufficient^32^ as compared with conventional microscopes. Vogelbaum et al^32^ performed a clinical trial on 20 patients with high-grade glioma,^32^ collecting 6 biopsies per patient from tissues with various fluorescence qualities (strong, weak, and none). They reported a sensitivity of 75%, specificity of 80%, PPV of 95%, and NPV of 39%. In a recent publication, others reported stronger fluorescence and background light intensities using the ORBEYE, but significantly more photobleaching^33^ than a microscope.

In a third study, the fluorescence mode was also reported to be superior to that of the microscope (7/7).^34^ However, 1 of 5 surgeons (20%) rated the handling of the fluorescence mode as worse than that when using a conventional microscope.

A general device disadvantage was that the wide-field illumination cone was not automatically adjusted to the observed field when zooming in, thus losing light intensity predominantly in deep cavities.

### Customized Surgical Loupe Systems

Several authors describe modifications to commercially available binocular loupes for FGR. Kuroiwa et al^35^ equipped HEINE HR loupes (Heine Optotechnik) with a semiconductor laser and transparent ultraviolet (UV) cutoff filter, which enabled FGR without on-off manipulation. The authors reported more distinct fluorescence in 1 patient, possibly allowing more precise resection.

Suero et al conducted a prospective, randomized, blinded validation study for a customized triple-LED/loupe device (REVEAL, Design for Vision Inc) for visualizing fluorescence.^18^ The triple-LED system featured a white light diode, 405-nm diode for excitation, and 450-nm diode for background illumination.^18^ Magnifying loupes contain a 475 longpass filter.^18^ The device was compared with the BLUE400 filter system (Kinevo 900, CZM).^18^ The comparison was based on fluorescence detection in freshly collected tissue specimens in a double-blind design,^18^ and statistical equivalence was observed. Measurements of fluorescence intensity with an “artificial eye” containing a power meter, placed behind the loupes or ocular of the microscope, revealed fluorescence and background illumination intensities to be approximately 10-fold compared with the microscope.^18^

In another study, Zhang et al^36^ performed superficial resections using the REVEAL FGS, describing more vivid fluorescence and finding similar neuropathological sensitivities and specificity compared with the microscope. However, no statistical analysis was presented.

A different headlamp system equipped with 2 LEDs, one emitting blue light (400-440 nm; peak 416 nm) and the other white light, was described by Woo et al.^37^ In 2 of 3 patients, initial resection was performed using a microscope, and later, the headlamp was used. In 1 of 3 patients, resection was initially performed with the headlamp. Fluorescent biopsies revealed tumor in all biopsies collected with the loupe system. The location of biopsies was not disclosed, and no statistical analysis was performed.

Gianti-Larsen and colleagues compared 3 low-cost microscope alternatives for FGR—a UV flashlight (Escolite UV flashlight Black Light, 51 LED, 395 nm UV), UV headlamp (EverBrite Black Light Headlamp UV, 395-400 nm purple light), and a REVEAL FGS system (Designs for Vision Inc).^38^ Using the UV headlamp for FGR, only approximately 90% of the resection could be performed; resection in deeper cavities required a microscope. The REVEAL system was used for 18 surgeries with rare switches to the microscope. Compared with the other 2 devices in this study, a better visualization was described using the REVEAL FGS headlamp, with no statistical analysis being reported.

Table 3 summarizes our findings regarding device evaluation as extracted from existing literature. Neuropathological validation in a truly reproducible way was rarely performed. Only one study addressed photobleaching. In addition, the influence of autofluorescence and possible false-positive results were not discussed.

**TABLE 3.** Assessment Criteria Defined for This Review to Evaluate Fluorescence Detection Devices

Devices	Fluorescence and background illumination intensity: subjective assessment	Assessment of photobleaching	Validation					Observer image and assistant integration
			Clinical validation	No. of patients	Diagnostic accuracy	Comparison with ES	Bias during tissue collection considered?	
Orbeye *Exoscope*	Quality of fluorescence illumination rated superior compared with OPMI.^31^	Yes^33^	Yes^31^	20	Truth standard: Pathology Specificity 80% Sensitivity 75% PPV 95% NPV 39%	No	No	3D 4K monitor
Aeos *Exoscope*	Mean fluorescence in OPMI greater than in the AEOS.^22^ OPMI superior for image contrast.^22^	No	Yes^22^	16	No tests on diagnostic accuracy performed.	Yes	Not performed	3D 4K monitor
Blue400 AR Filter system	Superior in the novel Blue400 AR filter compared with the conventional Blue400.^12^	No	Yes^12^	32	Truth standard: Pathology Specificity 97.37% Sensitivity 70.89% PPV 98.44% NPV 57.81%	Yes	Yes	Conventional microscope
HD-Xoscope *Exoscope*	N/A	No	Yes^28^	8	Truth standard: Pathology Specificity 100% Sensitivity 73%	No	No	23″ high-definition video monitor
REVEAL *Loupe system*	Superior fluorescence and background brightness.^18^	No	Yes^18^	26	Truth standard: BLUE400 Specificity 95% Sensitivity 100% PPV 98% NPV 100%	Yes	Not performed	Not possible
Storz Endoscope *Video camera*	N/A	No	Yes^25^	26	Specificity 75% Sensitivity 95.5%	No	No	Monitor
Fiberscope N-4L	Good discrimination of fluorescence.^17^	No^17^	No (single case)	1	N/A	No	N/A	Monitor
BLUE400	N/A	No	Yes^7^	33	Specificity 75% Sensitivity 70% PPV 98% NPV 28%	ES	Yes	Conventional microscope
Leica FL400	N/A	No	Yes^5^	69	Specificity 29.4% Sensitivity 96.5% PPV 95.4%	No	N/A	Conventional microscope

ES, established standard; NPV, negative predictive value; PPV, positive predictive value.

Assessment criteria defined for this review to evaluate fluorescence detection devices. Filter characteristics were not disclosed in all cases in the respective publications or by the manufacturer. This table is based on all articles identified in our research evaluating the respective devices.

## DISCUSSION

We here review studies on novel intraoperative fluorescence visualization devices, and it appears that standards for validating or testing such devices are lacking. While novel technologies can be expected to overcome limitations of the established standard, they should be validated to ensure they provide the well-tested selectivity of conventional systems, such as BLUE400, used for the 5-ALA approval studies.^3,7,39^ If more sensitive, novel systems would have to be carefully tested regarding tumor selectivity. Less sensitive systems should not be recommended further.

Confounders to be considered while testing are the handling of weak but omnipresent tissue autofluorescence and background illumination, which is necessary for giving tissue detail and might mask fluorescence if too strong.^6^ Furthermore, the influence of bleaching should be assessed in devices with strong illumination.

We have recently proposed general criteria for evaluating second-generation fluorochromes and devices with a focus on biases and confounders involved in such evaluations.^19^ We now find sources of bias in several publications. For example, studies on the Karl Storz endoscope/D-Light C device, which correlates fluorescence with pathology, described “infiltrating” tissues without providing a generally accepted definition of such tissues.^25,26^ Even in the exemplary study by Vogelbaum et al^32^ on the ORBEYE device, bias cannot be ruled out, despite the fact that a highly defined biopsy algorithm from different regions of fluorescence was followed. Going by the STARD-CNS guidelines, tissue allocation biases A and B were not considered, which the authors already partially acknowledged. It would have been of value to collect samples at a prespecified distance from the tumor margin, ie, to reach the infiltration zone and to use immunohistochemistry to identify infiltrating tumor cells more reliably.

Other possible biases involved in device validation by neuropathology might arise from differing biopsy frequency per patient, pooling samples from different patients, biases resulting from selecting a certain sensitivity threshold of an imaging device, timing bias if fluorochromes underlie time-dependent fluctuations in tissue after application, and biases resulting from methods for histological assessment.^19^

One relevant aspect rarely addressed in publications evaluating new devices is photobleaching.^2,4^ Photobleaching describes the destruction of a fluorophore during prolonged light exposure, possibly leading to loss of fluorescence and false-negative results.^4^ Using a standard microscope at a working distance of 25 cm, illumination decreases fluorescence to approximately 36% after approximately 27 minutes.^2^ The Orbeye was tested in this regard and had a much stronger bleaching effect than BLUE400.^33^ Different PpIX concentrations were excited with blue light using the Orbeye and Blue400 for up to 10 minutes.^33^ Analysis revealed lower fluorescence intensities using the Orbeye after continuous irradiation.^33^ In the margins of the resection cavity PpIX concentrations are low and continuous irradiation of these areas showed macroscopically significantly less fluorescence intensities.^33^

Regarding workflow, established FGR microscopes have disadvantages. Emitted fluorescence light is distributed foremost to the surgeon's oculars and complex camera systems and is barely perceived by the assistant. Using loupes, the primary surgeon will optimize his/her vantage point based on surgical necessity, often at the cost of the assistant's overview.^18^ Conversely, endoscopes and exoscopes create a screen image in 2D or 3D, the same for primary and assistant surgeons.^22,34,40^ Such workflow-related issues are rarely analyzed in available studies.

### Limitations

We here present a systematic review. Although we used a broad spectrum of search operators during our MEDLINE and Scopus search, some articles might not have been discovered in our search. Furthermore, novel devices are not immediately easily accessible to every clinic. Available data, therefore, merely represents a selected group of neurosurgical groups who evaluated these devices.

## CONCLUSION

As presented in this review, there are no current widely applied standards for validating and testing novel visualization devices for fluorescence visualization. In this respect, studies should be designed using prespecified end points and biometry; measures to reduce bias, such as rater blinding; and assessments of intraoperative evaluation by an independent review panel.^41^ If biopsies are collected, their exact location and size should be prespecified, as well as their actual numbers controlled for.^19^ Any neuropathological review should be standardized regarding the type of staining used, and raters should be unaware of fluorescence status.^19^

Because fluorescence recognition and stability can be influenced by several factors, neurosurgeons need to know whether devices show fluorescence equivalent to established standards. If more fluorescence is observed, histological corroboration of tumor selectivity is required. While new devices are marketed claiming to produce superior or comparable images, it is still being determined whether the diagnostic accuracy is similar to the established standard, whether photobleaching leads to worse sensitivity, and whether autofluorescence confounds fluorescence recognition. Future comparative and informative studies reflecting these points are warranted.

## SUPPLEMENTAL DIGITAL CONTENT

**Supplemental Figure 1.** Flow diagram of the study screening and selection process.
